# Gulf war toxicant-induced effects on the hippocampal dendritic arbor are reversed by treatment with a *Withania somnifera* extract

**DOI:** 10.3389/fnins.2024.1368667

**Published:** 2024-02-21

**Authors:** Amaan L. Shaikh, Kathleen E. Murray, Vijayalakshmi Ravindranath, Bruce A. Citron

**Affiliations:** ^1^Laboratory of Molecular Biology, Research & Development, Department of Veterans Affairs, VA New Jersey Health Care System, East Orange, NJ, United States; ^2^School of Graduate Studies, Rutgers University, Newark, NJ, United States; ^3^Centre for Brain Research, Indian Institute of Science, Bengaluru, India; ^4^Department of Pharmacology, Physiology, & Neuroscience, Rutgers New Jersey Medical School, Newark, NJ, United States

**Keywords:** Gulf War Illness, neurodegeneration, ayurveda, dendritic arborization, hippocampus, neuronal morphology, granule cells

## Abstract

Gulf War Illness (GWI) is a multi-symptom disorder that manifests with fatigue, sleep disturbances, mood-cognition pathologies, and musculoskeletal symptoms. GWI affects at least 25% of the military personnel that served in Operations Desert Shield and Desert Storm from 1990 to 1991. We modeled Gulf War toxicant exposure in C57BL/6J mice by combined exposure to pyridostigmine bromide (an anti-sarin drug), chlorpyrifos (an organophosphate insecticide), and DEET (an insect repellent) for 10 days followed by oral treatment with *Withania somnifera* root extract for 21 days beginning at 12 weeks post-exposure. *W. somnifera*, commonly referred to as ashwagandha, has been used in traditional Ayurvedic medicine for centuries to improve memory and reduce inflammation, and its roots contain bioactive molecules which share functional groups with modern pain, cancer, and anti-inflammatory drugs. Previously, we observed that GWI mice displayed chronic reductions in dendritic arbor and loss of spines in granule cells of the dentate gyrus of the hippocampus at 14 weeks post-exposure. Here, we examined the effects of treatment with *W. somnifera* root extract on chronic dendrite and spine morphology in dentate granule cells of the mouse hippocampus following Gulf War toxicant exposure. GWI mice showed approximately 25% decreases in dendritic length (*p* < 0.0001) and overall dendritic spine density with significant reductions in thin and mushroom spines. GWI mice treated with the Ayurvedic *W. somnifera* extract exhibited dendritic lengths and spine densities near normal levels. These findings demonstrate the efficacy of the Ayurvedic treatment for neuroprotection following these toxic exposures. We hope that the extract and the neuronal processes influenced will open new avenues of research regarding treatment of Gulf War Illness and neurodegenerative disorders.

## Introduction

Gulf War Illness (GWI) is recognized as a multi-symptom disorder that affects over 30% of the 700,000 military personnel that served in Operations Desert Shield and Desert Storm from 1990 to 1991 (Fukuda et al., 1998; Steele, 2000; RAC-GWI, 2014). Though the Gulf War was a military success, GWI is associated with many chronic health issues including fatigue, cognitive problems, pain, skin rashes, gastrointestinal problems, and respiratory difficulties (White et al., 2016). Affected Gulf War Veterans show an increased incidence of neurological impairments and often develop deficits in learning, memory, attention, and executive function (Lange et al., 2001; RAC-GWI, 2014; White et al., 2016). This cluster of symptoms is medically unexplained other than sharing a common etiology of chemical exposure (Gwini et al., 2016). The lack of information regarding the pathophysiology of GWI, among other factors, is a major obstacle to identifying effective interventions for afflicted Veterans (Freeman et al., 2020).

The unusual prevalence of Gulf War deployment-associated symptoms is thought to result from a combination of toxic exposures including agents that can affect acetylcholinesterase (RAC-GWI, 2014; White et al., 2016). Candidate toxicants include the pesticide chlorpyrifos (CPF), the nerve agent prophylactic pyridostigmine bromide (PB) (Chaney et al., 1997; Moss, 2001), insect repellent *N,N*-diethyl-*m*-toluamide (DEET), and possibly environmental stressors (Michalovicz et al., 2020). We previously reported acute transcriptional changes, detected by RNA-seq, in the mouse hippocampus following subcutaneous administration of a Gulf War toxicant mixture containing PB, CPF, and DEET (Murray et al., 2021). Gene ontology analysis found that genes related to dendritic spine development, development regulation, and spine morphogenesis were dysregulated. Gulf War toxicant-exposed mice displayed a 1.6-fold reduction in novel arm preference on a Y-maze task, suggesting impairment of hippocampal-dependent spatial reference memory. We also reported reductions in dendritic arbors and spine densities of dentate granule cells of the hippocampus following the same Gulf War toxicant insult (Murray et al., 2023).

*Withania somnifera*, commonly known as Ashwagandha, is a nootropic agent and Ayurvedic herb that was found to provide neurological benefits (Kulkarni and Dhir, 2008; Sehgal et al., 2012; Joshi and Joshi, 2021). *W. somnifera* is known to possess various withanolides and alkaloids which are thought to be the active compounds responsible for its bioactivity, such as Withaferin A, Withanolide A, and Withanoside IV (Mikulska et al., 2023). Administration of *W. somnifera* extract for 30 days was shown to reduce cognitive deficits and reverse disease pathology in a mouse model of Alzheimer’s disease (AD) (Sehgal et al., 2012). Withaferin A inhibits amyloid-β production as well as the gene expression of neuroinflammation molecules related to NF-κB (Bhargava et al., 2019; Mikulska et al., 2023). Neuronal degeneration and death in AD may be caused by the accumulation of Aβ plaques and neurofibrillary tangles made of hyperphosphorylated tau proteins, which lead to axonal atrophy, synaptic collapse, and neuronal degeneration. This may explain the effectiveness of *W. somnifera* as a treatment of AD as well as support the use of *W. somnifera* for neuroprotection and for treatment in other neurodegenerative conditions.

In this study, we examined the therapeutic effect of *W. somnifera* root extract on the dendritic morphology of dentate granule cells in the hippocampi of male and female mice exposed to the GWI-related toxicants PB, CPF, and DEET (Murray et al., 2021). We tested the extract based on experience with other models of neurodegeneration (Sehgal et al., 2012; Saykally et al., 2017), to determine whether it would also be effective in treating certain aspects of GWI. We measured dendritic lengths, spine counts, and spine densities. Our results suggest that the damaging effects of GW toxicants on neuronal connectivity can be improved by treatment with *W. somnifera* root extract.

## Materials and methods

### Subjects

Male C57BL/6J mice (11 weeks old) were obtained from Jackson Laboratory (000664, Bar Harbor, ME, USA). Mice were group housed in a 22 ± 0.5°C temperature-controlled environment with a 12-h light/dark cycle and allowed a 7 day acclimation period prior to handling. Food and water were available *ad libitum* for all animals. All experiments were performed according to the guidelines of the institution and the National Research Council’s Guide for the Care and Use of Laboratory Animals and approved by the VA New Jersey Institutional Animal Care and Use Committee.

### Gulf war toxicant exposure

**FIGURE 1:**
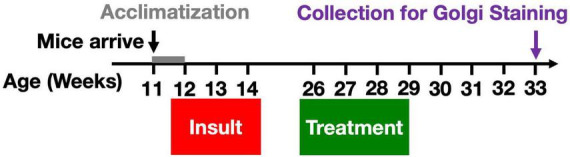
Gulf War toxicant exposure model. A total of 12-week-old male and female C57Bl/6J mice received daily s.c. injections of either toxicant mixture containing PB (0.525 mg/kg), CPF (9.38 mg/kg), and DEET (5.63 mg/kg), or vehicle for 10 days (Mon-Fri for 2 weeks). Twelve weeks after the final insult, the mice were treated with *W. somnifera* root extract (1 g/kg) via oral gavage for 3 weeks. Four weeks later, brain tissue was harvested for use in Golgi staining in order to characterize chronic effects on dendritic length and spine densities by subtype.

HPLC-grade pyridostigmine bromide (PB, P9797), N,N-diethyl-m-toluamide (DEET, D100951), dimethyl sulfoxide (DMSO, 99.9%, D2438), and chlorpyrifos (CPF, N-11459) were purchased from Sigma-Aldrich (St. Louis, MO, USA). The toxicant mixture stock solution was stored in aliquots at –20°C and diluted in 1X PBS before injections. Injection vehicle contained 3.125% DMSO in PBS. Mice [*n* = 6–8 (males + females)/group] received daily subcutaneous (s.c.) injections of either Gulf War toxicants (0.525 mg/kg PB, 9.38 mg/kg CPF, and 5.63 mg/kg DEET with 3.125% DMSO in PBS) or vehicle (3.125% DMSO in PBS) for 10 days (Mon-Fri for 2 weeks) starting at 12 weeks old (Figure 1). Dosages were based on previous work (Torres-Altoro et al., 2011; Ojo et al., 2014; Murray et al., 2021). Animals were monitored for adverse effects during the insult phase, e.g., for excessive weight loss or seizures. Our experience with these toxicants indicates a very steep toxicity curve (that may be sensitive to mouse cohort, environment, and diet) with viability in jeopardy at overall dosing beginning 25% higher. Most mice exhibited mild, brief tremor-like behavior shortly after injections.

### Treatment

Mice received *W. somnifera* root extract (1 g/kg bodyweight) in 5% ethanol or vehicle via oral gavage once daily for 21 days [*n* = 6–8 (males + females)/group] beginning 12 weeks after the end of the toxicant exposure (Figure 1). The dosing for the treatment with *W. somnifera* extract that we administered in this study was the same as in the AD study, a characterized serial extraction from powdered plant root, containing 75% withanolides and 20% withanosides which has been described previously (Sehgal et al., 2012). *W. somnifera* was prepared weekly for dosing by suspending the extract paste in ethanol at 150 mg/mL such that no animal received more than 0.3 mL via gavage.

### Golgi staining

Golgi staining was conducted using the FD Rapid GolgiStain Kit (PK104, FD NeuroTechnologies, Columbia, MD, USA) to assess chronic morphological properties of granule cell dendrites in the dentate gyrus of the hippocampus at 33 weeks of age (19 weeks post-exposure). Mice were anesthetized with isoflurane and euthanized via cervical dislocation and decapitation, and fresh whole brains were extracted. Brains were briefly rinsed with ultrapure water to remove any excess blood from the surface and immediately placed in the impregnation solution (Solution A+B) for 1 days per manufacturer instructions. Brains were switched to transport solution (Solution C) and shipped to NeuroDigiTech (San Diego, CA, USA) for completion of the staining procedure and analysis. Coronal sections, 120 μm thick, were collected with a vibratome. Six dentate gyrus granule cells per brain and at least five (male + female) brains per group were evaluated.

### Dendritic morphology and spine assessment

Dentate gyrus cells were characterized for morphological changes in dendritic arborization and spine density by digital reconstruction using a commercially available stereology software program (Neurolucida, MBF Bioscience, VT, USA). Slides were made of brains from samples that included the basal dendrites of dentate gyrus cells and analyzed with the aid of Neurolucida software and a Nikon Eclipse Ni microscope with a high-resolution CCD camera. To select samples, coronal sections along the entire rostro-caudal axis were previewed under low magnification (10x and 20x), with regions containing the least truncations on distal dendrites selected using higher magnification (40x and 63x). A 3D dendritic reconstruction was performed (Zeiss 100x with immersion oil) to count spines in the dendritic trees of selected neurons. Candidate neurons were chosen based on how well the soma was visualized (having no overlap with neighboring soma or incomplete impregnation with Golgi silver), and whether the 3D profile of the dendritic trees could be completely visualized via the imaging software. For dendritic spine sampling, only spines orthogonal to the dendrite were included as they were the only spines that could be readily resolved.

### Statistics

Group means of total dendritic length, overall spine density, total spine counts, and spine density of thin and mushroom spines were analyzed using a nested one-way ANOVA and Tukey’s multiple comparison test. Graphs depict group means ± standard error (*p < 0.05, **p < 0.01, ***p < 0.001). Statistical analyses were performed with Prism for macOS (version 10.1, GraphPad Software, San Diego, CA, USA).

## Results

### Dendritic lengths in dentate granule cells are reduced by gulf war toxicant exposure and recovered by treatment with *W. somnifera* in male and female mice

**FIGURE 2:**
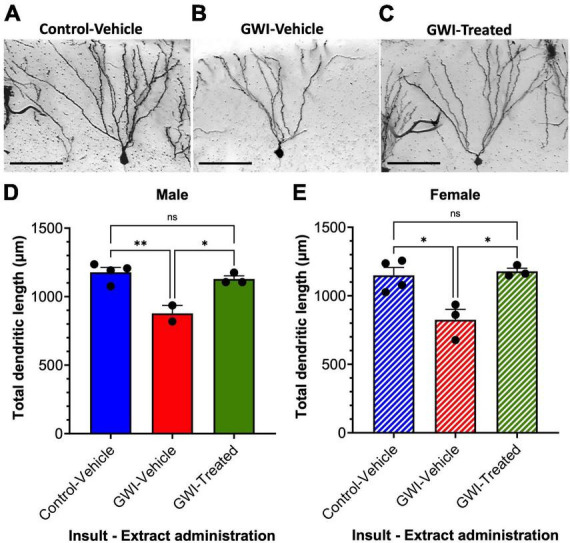
Dendritic architecture and lengths of granule cells in the dentate gyrus. Micrographs of representative hippocampal granule cells at 19 weeks post-exposure to **(A)** vehicle insult and control treatment **(B)** PB + CPF + DEET with no treatment, or **(C)** PB + CPF + DEET with *W. somnifera* treatment via oral gavage. Scale bars: 50 μm. **(D,E)** Total dendritic length (μm) per neuron was reduced in GWI mice but improved with treatment in both male and female mice (19 total mice; *p < 0.05, **p < 0.01).

Golgi staining was performed to assess dendritic length at 19 weeks post-exposure to PB + CPF + DEET after 21 days of treatment with *W. somnifera*. A micrograph of representative hippocampal neurons highlights the loss of dendritic arborization in Gulf War toxicant-exposed mice (Figures 2A, B) followed by the rescue of the dendritic arbors after treatment (Figure 2C). Total dendritic length in males was reduced by 26% in GWI-Vehicle mice (Control-Vehicle: 1178 ± 35.12 μm, GWI-Vehicle: 877.5 ± 58.5 μm, *p* = 0.0040) but increased by 29% due to *W. somnifera* treatment (GWI-Treated: 1129 ± 23.0 μm, *p* = 0.012). There was no significant difference between dendritic lengths of male Control-Vehicle and GWI-Treated mice (Figure 2D). Dendritic length in females was similarly reduced by 28% in GWI-Vehicle mice (Control-Vehicle: 1149 ± 57.3 μm, GWI-Vehicle: 824 ± 76.7 μm, *p* = 0.0121) but increased by 43% by *W. somnifera* treatment (GWI-Treated: 1178 ± 23.35 μm). There was also no significant difference between lengths of Control-Vehicle and GWI-Treated female mice (Figure 2E).

### *W. somnifera* treatment recovers overall spine density of dentate granule cells in male and female GWI mice

**FIGURE 3:**
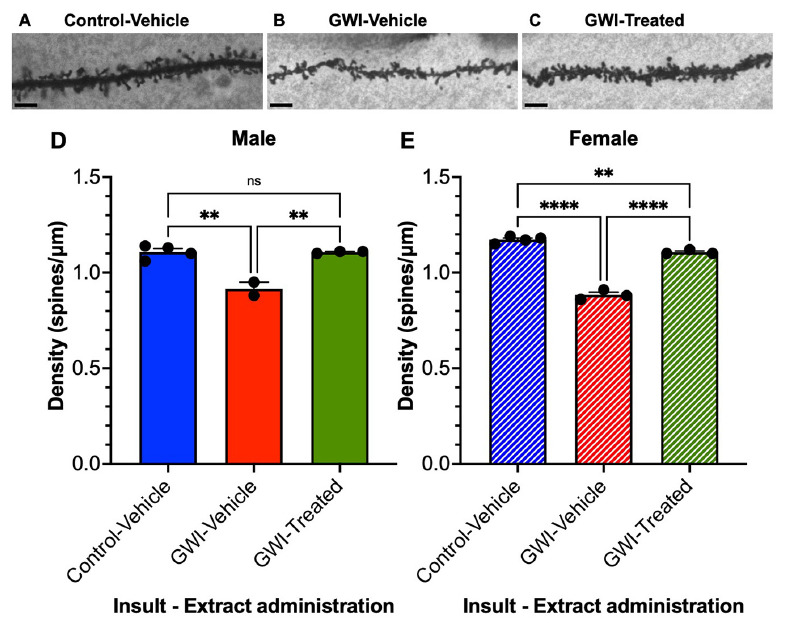
Overall spine density of granule cells in the dentate gyrus. Micrographs of representative dendritic spines of granule cells at 19 weeks post-exposure to **(A)** vehicle with no treatment, **(B)** PB + CPF + DEET with no treatment, or **(C)** PB + CPF + DEET with *W. somnifera* treatment via oral gavage. Scale bars: 5 μm. **(D,E)** Total dendritic spine density (μm) decreased in GWI mice but treatment rescued this in both male and female mice (19 total mice; **p < 0.01, ****p < 0.0001).

Dendritic spine densities were determined from the dentate gyrus granule cells (19 weeks post-exposure to PB + CPF + DEET after 21 days of treatment with *W. somnifera*) (Figures 3A–E). Spine density was recorded as the number of spines per μm, determined throughout the dendritic arbors. Micrographs of representative spines are shown of Control and GWI mice (Figures 3A, B) followed by a representative spine that was exposed and treated with the extract (Figure 3C). Overall spine density in males was reduced by 17% due to GW toxicant exposure (Control Vehicle: 1.108 ± 0.0180 spines/μm, GWI-Vehicle: 0.915 ± 0.035 spines/μm, *p* = 0.0012) but increased by 21% with treatment (GWI-Treated: 1.107 ± 0.0033 spines/μm, *p* = 0.0016). There was no significant difference between Control-Vehicle and GWI-Treated mice (Figure 3D). Spine density in female mice was reduced by 25% (Control Vehicle: 1.173 ± 0.00854 spines/μm, GWI-Vehicle: 0.883 ± 0.0145 spines/μm, *p* < 0.0001) and increased another 25% from there with treatment (GWI-Treated: 1.107 ± 0.00667 spines/μm, *p* < 0.0001). However, overall spine density in the female mice was still 6% lower in the treated group than the Control-Vehicle (*p* = 0.0068) (Figure 3D).

### *W. somnifera* treatment enhances total spine counts in male and female GWI mice

**FIGURE 4:**
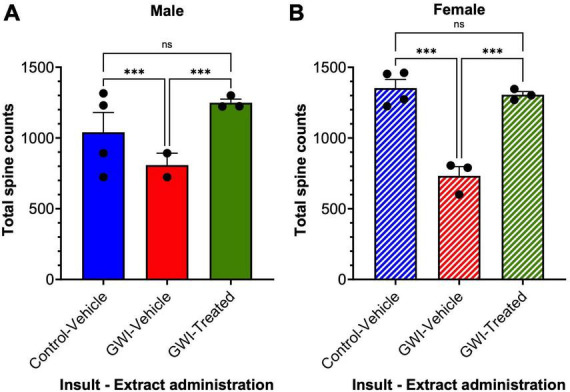
Spine counts of granule cells in the dentate gyrus. Golgi staining of dendritic spines of granule cells at 19 weeks post-exposure to vehicle with no treatment, PB + CPF + DEET with no treatment, or PB + CPF + DEET with *W. somnifera* treatment via oral gavage. **(A,B)** Dendritic spine counts decreased with exposure to GWI toxicant mixture, but significantly improved with administration of extract (19 total mice; ***p < 0.001).

Total spine counts displayed more dramatic differences. Total spine counts in male mice were reduced by 38% after toxicant exposure (Control-Vehicle: 1302 ± 24.52 μm, GWI-Vehicle: 808.1 ± 84.73 μm, *p* = 0.0003), with an increase of 55% after *W. somnifera* treatment (GWI-Treated: 1249 ± 25.74 μm, *p* = 0.0008). There was no significant difference between Control-Vehicle and GWI-Treated groups (Figure 4A). In female mice, spine counts decreased by 46% (Control-Vehicle: 1353 ± 60.82 μm, GWI-Vehicle: 732.2 ± 66.03 μm, *p* = 0.0002), and treatment caused a dramatic 78% increase (GWI-Treated: 1306 ± 22.43 μm, *p* = 0.0006). There was also no significant difference between the treated and control groups in the female mice in terms of spine counts (*p* = 0.825) (Figure 4B).

### Thin and mushroom spine densities in dentate granule cells are reduced by gulf war toxicant exposure and improved by treatment with *W. somnifera* in male and female mice

**FIGURE 5:**
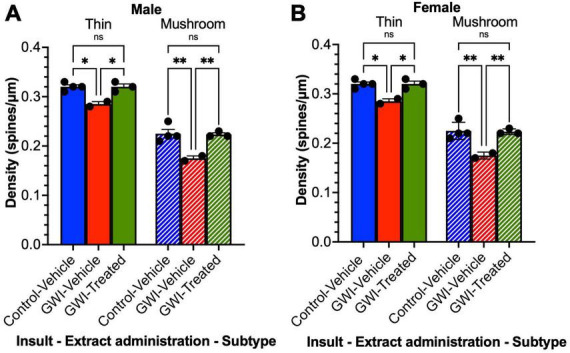
Spine density by subtype of granule cells in the dentate gyrus. Golgi staining of dendritic spines of granule cells at 19 weeks post-exposure to vehicle with no treatment, PB + CPF + DEET with no treatment, or PB + CPF + DEET with *W. somnifera* treatment via oral gavage. **(A,B)** Both thin and mushroom subtype spine densities were detrimentally affected in GWI mice, but treatment enhanced them significantly (19 total mice; *p < 0.05, **p < 0.01).

The density of different subtypes of dendritic spines of the sampled hippocampal neurons was also quantified for thin and mushroom type spines. For the males, thin spine density decreased with toxicant exposure by 11% after exposure (Control-Vehicle: 0.32 ± 0.0041 spines/μm, GWI-Vehicle: 0.285 ± 0.005 spines/μm, *p* = 0.0085), and a 12% increase in density was observed after treatment (GWI-Treated: 0.32 ± 0.0058 spines/μm, *p* = 0.012). There was no significant difference between treated and control male thin spine densities. Similarly with male mushroom spines, there was 22% drop in density (Control-Vehicle: 0.225 ± 0.0087 spines/μm, GWI-Vehicle: 0.175 ± 0.005 spines/μm, *p* = 0.0006), with a 27% improvement observed following *W. somnifera* treatment (GWI-Vehicle: 0.223 ± 0.0033 spines/μm, *p* = 0.0012). There was no significant difference between GWI-Treated and Control-Vehicle mushroom densities in males (Figure 5A). Similar results were observed in the females (Figure 5B). Thin spine density decreased by 11% (Control-Vehicle: 0.32 ± 0.00408 spines/μm, GWI-Vehicle: 0.285 ± 0.005 spines/μm, *p* = 0.0303) and increased by 12% with *W. somnifera* administration (GWI-Treated: 0.32 ± 0.0058 spines/μm, *p* = 0.0416). There was no significant difference between Control-Vehicle and GWI-Treated densities. The findings for the mushroom subtype of spines were comparable, with a 22% decrease in density upon GWI toxicant exposure (Control-Vehicle: 0.225 ± 0.0087 spines/μm, GWI-Vehicle: 0.175 ± 0.005 spines/μm, *p* = 0.0023), and an increase of 28% after administering *W. somnifera* (GWI-Treated: 0.2233 ± 0.003 spines/μm, *p* = 0.0046). There was no significant difference between control and treated mushroom spine densities.

## Discussion

In this study, we found that exposure to a mixture of PB + CPF + DEET negatively affected dendritic arborization and architecture in neurons of the dentate gyrus of the hippocampus of both male and female mice. Specifically, we observed a decrease in dendritic length, overall spine counts, spine density, and densities of thin and mushroom spines. These effects were significantly improved by oral administration of *W. somnifera* root extract, which contains components such as withanolides and withanosides which are thought to have neuroprotective, antioxidant, and anti-inflammatory effects. These findings reinforce the idea that long-term effects to dendritic morphology occur after toxicant exposure, and that *W. somnifera* root extract can ameliorate chronic reductions in dendritic length, spine counts, and spine density.

Various Gulf War toxicant exposure models have demonstrated detrimental effects on the hippocampus. Exposure to DEET, PB, permethrin (a non-organophosphate insecticide), and stress in rats was shown to significantly decrease hippocampal volume and neuron growth, as well as cause memory problems and lower spatial learning (Parihar et al., 2013). In another rat model, DEET and permethrin exposure caused cytoskeletal changes in the hippocampus, specifically in neurons of the dentate gyrus (Abdel-Rahman et al., 2001). CPF on its own or with combination with PB and permethrin decreased synaptophysin in the CA3 region of the hippocampus, which is important for memory encoding and retrieval (Ojo et al., 2014). PB and permethrin exposure increased astrogliosis and decreased synaptophysin expression in the hippocampus and cortex of mice at 5 months post-exposure (Zakirova et al., 2015). Exposure to DEET, PB, permethrin, and stress in a mouse model yielded an increase in astrocytosis which is significant because astrocytes are important for synaptic maintenance and postsynaptic density (Abdullah et al., 2012).

In addition to detrimental effects on hippocampal plasticity, GWI exposure is also known to lead to deficits in hippocampal learning and memory. We previously reported deficits in spatial memory on the Y-maze task with the GWI mouse model (Murray et al., 2021). Deficits in hippocampal-dependent object location memory and spatial memory in Morris water maze have also been described (Hattiangady et al., 2014). Veterans with GWI also had deficits in face-name associative recall [another test of hippocampal function, and deficits observed through fMRI analysis (Odegard et al., 2013; Cooper et al., 2016)]. Our results agree with and extend these findings as well as illustrate a clear degeneration in morphology of hippocampal dendritic arbors in response to Gulf War toxicant exposure.

*Withania somnifera* or Ashwagandha is a plant cultivated in Indian Ayurvedic medicine for its ability to rejuvenate and promote longevity of life (Kulkarni and Dhir, 2008; Sehgal et al., 2012). Many pharmacological studies have been performed to characterize the different responses to treatment with *W. somnifera* extract. Oxidative stress that can affect mitochondria is a characteristic of Gulf War exposures (Chen et al., 2017; Shetty et al., 2017; Delic et al., 2021). Rats that received intraperitoneal injection of *W. somnifera* root extract after rotenone-induced oxidative stress had similar levels of antioxidants and lipid peroxidation to non-stress controls, demonstrating the anti-oxidative properties of *W. somnifera* (Epuri et al., 2023). In a rat chronic unpredictable stress (CUS) model, an Ashwagandha sustained release formulation improved spatial memory with a Morris water maze test and measures of anxiety in an elevated plus maze test (KrishnaRaju et al., 2023). These studies help to substantiate *W. somnifera* as an adaptogen or stress attenuator, as Ashwagandha is commonly marketed for this purpose. In the context of GWI, stress (in addition to the acetylcholinesterase inhibitors and toxicants) is a common denominator for many Veterans and that *W. somnifera* treatment has an impact in a stress model bolsters its stock as a potential avenue for therapeutic development.

*Withania somnifera* has also been investigated for its role in treating neurodegenerative diseases. *W. somnifera* improved cognition as well as plaque and β-amyloid peptide accumulation in the APP/PS1 mouse model of AD (Sehgal et al., 2012). This supports *W. somnifera* as a promising treatment for GWI, since recent studies have suggested tau accumulation as one factor leading to cognitive problems and neurodegeneration based on observations of tau autoantibodies in the blood of afflicted Veterans (Baas et al., 2023). In a Parkinson’s disease (PD) mouse model, treatment with *W. somnifera* was able to protect dopaminergic neurons from apoptosis by modulating oxidative stress and reducing GFAP expression, a marker of astrocyte activation (Prakash et al., 2014). Many of these effects are related to the activity of withanolides and withanosides present in the root extract of *W. somnifera*. Withanolide A was found to prevent neurodegeneration caused by hypoxia by increasing glutathione biosynthesis in the hippocampus by upregulating γ-glutamate-cysteine ligase (GCLC) through the Nrf2 pathway (Baitharu et al., 2014). In a human neuroblastoma SH-SY5Y cell line, withanolide A, withanoside IV, and withanoside VI contributed to statistically significant neurite outgrowth at a 1 μM concentration (Zhao et al., 2002). In rats, withanolide A was sufficient to induce regeneration of axons and dendrites in the hippocampus and cerebral cortex of Aβ treated cells as well as recover a memory deficit (Kuboyama et al., 2005). In mice, oral withanoside IV improved memory deficits in Aβ-injected mice and prevented loss of dendritic architecture, and sominone (a withanoside IV metabolite) had similar, protective effects (Kuboyama et al., 2006). These findings are consistent with our results, where administration of *W. somnifera* to mice with neurodegeneration from toxicant exposure dramatically improved and repaired dendritic architecture.

## Conclusion

This study focused on changes to dendritic morphology, specifically dendritic length and spine density and subtype in a Gulf War model. Dendritic arbors of GWI mice (both male and female) displayed statistically significant reductions of total dendritic length, total spine count, overall spine density and density by subtype (thin and mushroom). Treatment of GWI mice with *W. somnifera* returned dendritic length, spine count, and spine density overall and by subtype to levels similar to controls. These findings suggest that *W. somnifera* is a promising avenue for further research on treating aspects of Gulf War Illness, as well as other neurodegenerative diseases. Additional studies into evaluations of cognitive and memory functions following treatment are also warranted. Future studies in the hippocampi of GWI-exposed mice may shed light on potential neuroplasticity effects, and additional insight into mechanistic changes.

## Data availability statement

The raw data supporting the conclusions of this article will be made available by the authors, without undue reservation.

## Ethics statement

The animal study was approved by the VA New Jersey Health Care System Institutional Animal Care and Use Committee. The study was conducted in accordance with the local legislation and institutional requirements.

## Author contributions

AS: Data curation, Formal analysis, Writing – original draft. KM: Formal analysis, Investigation, Methodology, Writing – review and editing. VR: Conceptualization, Formal analysis, Investigation, Resources, Writing – review and editing. BC: Conceptualization, Formal analysis, Funding acquisition, Investigation, Methodology, Project administration, Writing – review and editing.
