# Correction: Gulf war toxicant-induced effects on the hippocampal dendritic arbor are reversed by treatment with a *Withania somnifera* extract

**DOI:** 10.3389/fnins.2025.1722602

**Published:** 2025-11-18

**Authors:** Amaan L. Shaikh, Kathleen E. Murray, Vijayalakshmi Ravindranath, Bruce A. Citron

**Affiliations:** 1 Laboratory of Molecular Biology, Research & Development, Department of Veterans Affairs, VA New Jersey Health Care System, East Orange, NJ, United States; 2 School of Graduate Studies, Rutgers University, Newark, NJ, United States; 3 Centre for Brain Research, Indian Institute of Science, Bengaluru, India; 4 Department of Pharmacology, Physiology, & Neuroscience, Rutgers New Jersey Medical School, Newark, NJ, United States

**Keywords:** gulf war illness, neurodegeneration, ayurveda, dendritic arborization, hippocampus, neuronal morphology, granule cells

There was a mistake in Figures 3A–C as published. Incorrect representative images were provided. There are no other changes besides replacing those three images. There are no changes to any text, no changes to the figure caption, and no changes to anything else in the figure. The corrected Figure 3 appears below.

**Figure 3:**
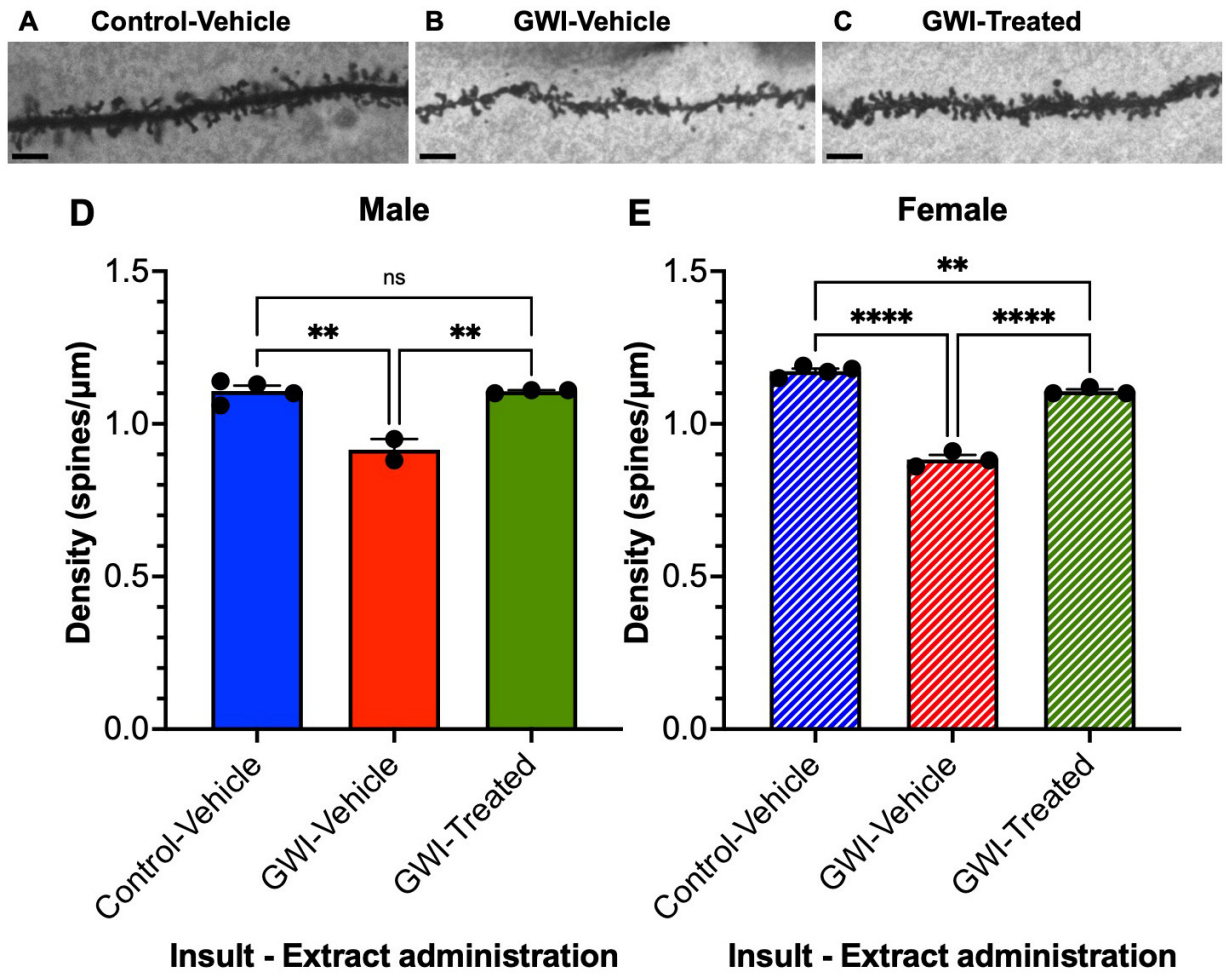
Overall spine density of granule cells in the dentate gyrus. Micrographs of representative dendritic spines of granule cells at 19 weeks post-exposure to **(A)** vehicle with no treatment, **(B)** PB + CPF + DEET with no treatment, or **(C)** PB + CPF + DEET with *W. somnifera* treatment via oral gavage. Scale bars: 5 μm. **(D, E)** Total dendritic spine density (μm) decreased in GWI mice but treatment rescued this in both male and female mice (19 total mice; ^**^p < 0.01, ^****^p < 0.0001).

The original version of this article has been updated.

